# Cyclodextrin-Appended Superparamagnetic Iron Oxide Nanoparticles as Cholesterol-Mopping Agents

**DOI:** 10.3389/fchem.2021.795598

**Published:** 2021-11-18

**Authors:** Antonino Puglisi, Simone Bassini, Erik Reimhult

**Affiliations:** ^1^ Department of Nanobiotechnology, Institute of Biologically Inspired Materials, University of Natural Resources and Life Sciences (BOKU), Vienna, Austria; ^2^ Life Sciences Department, University of Modena and Reggio Emilia, Modena, Italy

**Keywords:** cholesterol, cyclodextrin, SPION, core-shell nanoparticles, nanoparticles

## Abstract

Cholesterol plays a crucial role in major cardiovascular and neurodegenerative diseases, including Alzheimer’s disease and rare genetic disorders showing altered cholesterol metabolism. Cyclodextrins (CDs) have shown promising therapeutic efficacy based on their capacity to sequester and mobilise cholesterol. However, the administration of monomeric CDs suffers from several drawbacks due to their lack of specificity and poor pharmacokinetics. We present core-shell superparamagnetic iron oxide nanoparticles (SPIONs) functionalised with CDs appended to poly (2-methyl-2-oxazoline) polymers grafted in a dense brush to the iron oxide core. The CD-decorated nanoparticles (CySPIONs) are designed so that the macrocycle is specifically cleaved off the nanoparticle’s shell at a slightly acidic pH. In the intended use, free monomeric CDs will then mobilise cholesterol out of the lysosome to the cytosol and beyond through the formation of an inclusion complex. Hence, its suitability as a therapeutic platform to remove cholesterol in the lysosomal compartment. Synthesis and full characterization of the polymer as well as of the core-shell SPION are presented. Cholesterol-binding activity is shown through an enzymatic assay.

## Introduction

Cholesterol is a major component of cell membranes, and it plays an essential role in ordinary neuronal physiology (Simons and Gerl, 2010). Alterations in cholesterol’s metabolism are linked to severe neurological syndromes, such as Alzheimer’s disease, Huntington’s disease, and Parkinson’s disease, and some rare hereditary diseases (Martín et al., 2014). Innovative pharmacological approaches aiming at counteracting cholesterol imbalance, particularly in the brain, are therefore investigated. In such a context, cyclodextrins (Davis and Brewster, 2004) (CDs) and their derivatives are emerging as promising therapeutic tools in the treatment of cholesterol-associated vascular and neurodegenerative diseases (Camilleri et al., 1994; Yao et al., 2012; Coisne et al., 2016) as well in the treatment of Niemann-Pick disease Type C (NPC) (Liu, 2012; Calias, 2017; Hastings et al., 2019; NeßlauerAnna-Maria et al., 2019). In particular, based on its cholesterol-extracting action, a chemical modification of the seven membered-ring of the series (βCD), the 2-hydroxypropyl-β-cyclodextrin (HPβCD) is currently in phase I/II [NCT03893071] and phase II/III [NCT03893071] clinical trials for NPC treatment. Although promising, CD treatments have significant shortcomings, mainly due to poor pharmacokinetics and bioavailability (Loftsson et al., 2016), particularly in the brain (Calias, 2017). Various CD-based macromolecular systems have been suggested to be more efficient cholesterol-mopping therapeutic agents by reducing systemic clearance. In some cases, they have increased NPC mice’s life span in comparison to HPβCD (Kulkarni et al., 2018; Tamura and Yui, 2018; Puglisi and Yagci, 2019; Carradori et al., 2020). Nanoparticle formulations can improve the bioavailability of biologics and other macromolecular drugs (Patra et al., 2018). Superparamagnetic oxide nanoparticles (SPIONs) are currently a hot topic in nanomedicine because of their biocompatibility and ease of functionalization as well as their potential applications in both cancer therapy (Shi et al., 2017) and imaging (Kunjachan et al., 2015). SPIONs are good candidates for both targeting purposes and conjugation with biotherapeutics, as they can accumulate and remain within tissues for long and still undergo biocompatible degradation (Mok and Zhang, 2013). The use of SPIONs over other nanoparticle delivery platforms offers great advantages in terms of stability and surface modification. The decoration of SPIONs with therapeutic agents has shown superior therapeutic effects due to a decreased clearance rate, improved bioavailability, the possibility to target tissue, and the ability to image the distribution of the drug through the particle core (Wahajuddin and Arora, 2012). These advantages motivate us to develop SPIONs as a novel platform to deliver CDs, potentiating their therapeutic efficacy towards neurodegenerative disorders associated with cholesterol.

**GRAPHICAL ABSTRACT:**
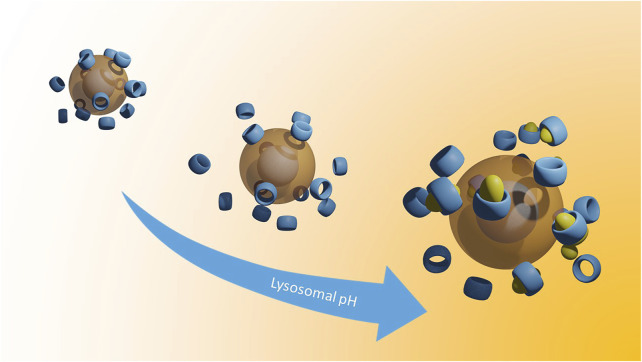


### Design Strategy

We propose a novel class of Cyclodextrin-decorated SPIONs (CySPIONs) tailored to treat cholesterol-impaired diseases. In our CySPION, βCDs are appended onto the SPION’s polymeric shell through bespoke chemistry that triggers the detachment of the macrocycle at slightly acidic pHs. By this, we aim to target the lysosomal subcellular compartment where the build-up of cholesterol occurs. The packaging of CDs into a nanoparticle offers a two-fold advantage over the administration of monomeric CDs. First, the increased size suppresses renal clearance and thereby improves circulation and the likelihood of endosomal targeting (Gould and Scott, 2005). Second, hydrophilic nanoparticles have been suggested to enhance passage through the Blood-Brain-Barrier (BBB). The BBB represents the main obstacle for delivering CDs into the brain. Specifically, SPIONs have shown transport across the BBB (Thomsen et al., 2013; Busquets et al., 2015; Shi et al., 2016).

**FIGURE 1:**
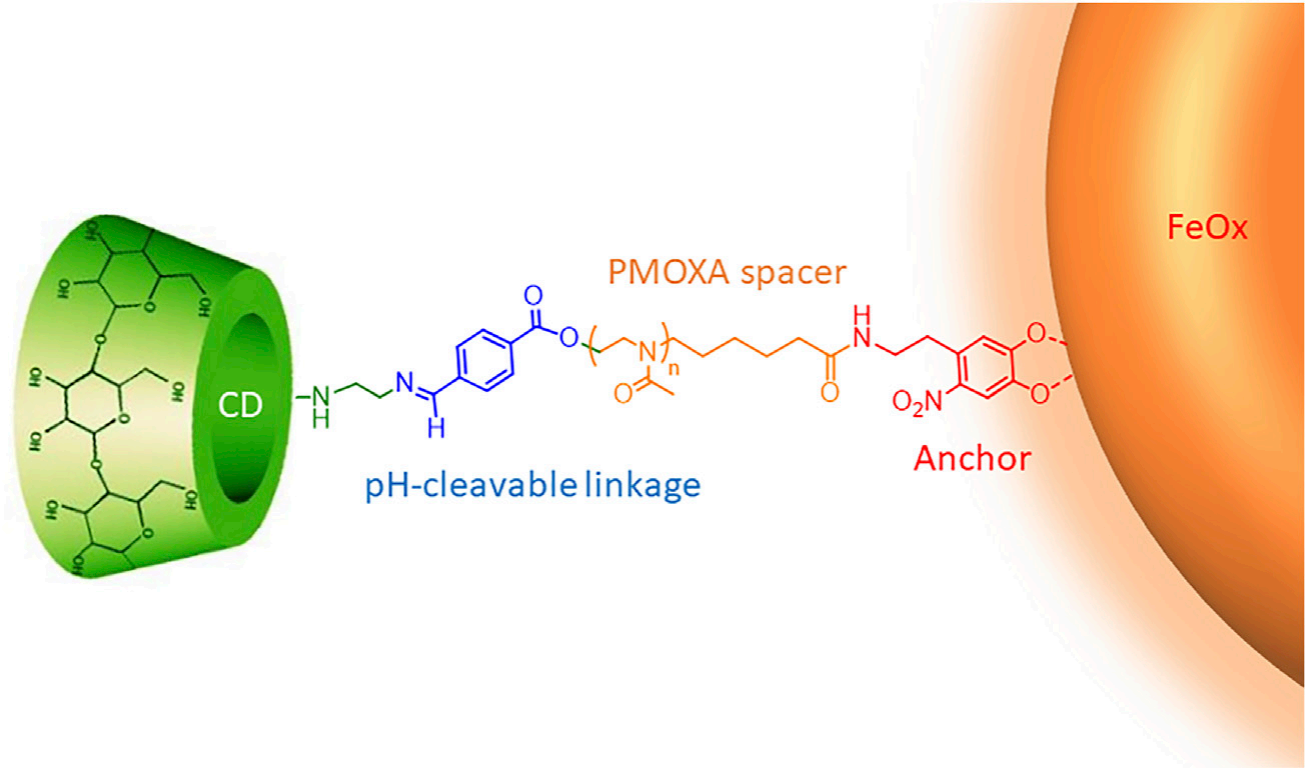
Schematic representation of the CySPION’s nano-architecture.

The CySPION’s nano-architecture (Figure 1) consists of a superparamagnetic iron oxide nanoparticle core coated with bi-functional poly (2-methyl-2-oxazoline) (PMOXA) polymers through a nitrodopamide anchor. Nitrodopamine was selected as the anchor as it has been reported to complex large polymer ligands at high grafting density irreversibly to iron oxide nanoparticles under physiological conditions (Zirbs et al., 2015). The PMOXA forms a dense but highly hydrated polymer brush that colloidally stabilises the particle and shields it from non-specific protein adsorption and biological response (Konradi et al., 2012; Morgese et al., 2018). We choose PMOXA because it has similar properties for biomedical purposes as the broadly used poly (ethylene glycol) (Chen et al., 2014; Victor, 2014). Still, it has additional advantages such as being easier to synthesise and functionalise and no demonstrated immunogenicity.

The βCD decoration is achieved through a pH-cleavable, benzoic imine functionality (Qu and Yang, 2016) embedded into the polymer structure. Similar approaches have already been followed for micellar systems (Zhou et al., 2016; Puglisi et al., 2019) and in drug delivery (Zan et al., 2014; Yu et al., 2015).

## Materials and Methods

All chemicals, unless otherwise specified, were purchased from Sigma-Aldrich and used as received without further purification. 2-Methyl-2-oxazoline was dried over CaH_2_ and distilled before use.

NMR spectra were collected on a Bruker Avance III HD 300 MHz using d6-DMSO as solvent and TMS as an internal standard and processed with Bruker Topspin 3.5 PL six software.

TEM studies were performed on an FEI Tecnai G2 20 transmission electron microscope operating at 120 or 200 kV for high-resolution imaging. Samples were prepared by dropping toluene dispersions of oleic acid-coated iron oxide core nanoparticles onto a 300-mesh carbon-coated copper grid and subsequently evaporating the solvent in air.

Thermogravimetric Analysis (TGA) and Differential Scanning Calorimetry (DSC) measurements. Thermograms were recorded on a Mettler-Toledo TGA/DSC 1 STAR system in the temperature range 25–650°C with a ramp of 10 K/ min in a synthetic air stream of 80 ml/ s to ensure complete combustion of ligands as NDA was found to polymerize by pyrolization under N_2_. 70 μL aluminum oxide crucibles were filled with 0.5–1.5 mg of sample, and the total organic content (TOC) was evaluated as the mass loss fraction at 500°C by horizontal setting.

IR spectra were obtained with an FT-IR ATR spectrometer (Vertex 70, Bruker, Billerica, United States ) with 16/32 scans. The samples were directly mounted on the ATR unit and measured with the pressure stamp. Five measurements were averaged, cut, and baseline corrected using OPUS 7.5 software (Bruker, United States )

### Synthesis of Oleic Acid-Coated Iron Oxide Cores

Oleic acid (OA)-stabilized superparamagnetic magnetite nanoparticles were synthesized by thermal decomposition of an iron pentacarbonyl precursor as previously described (Lassenberger et al., 2016). Briefly, a mixture of 50 ml of dioctyl ether and 5.5 ml of OA was heated to 100°C under nitrogen. 1.1 ml of Fe(CO)_5_ was rapidly injected, and the reaction mixture was heated to 290°C with a temperature ramp of 3°C/ min. After aging for 1 h, the NP dispersion was allowed to cool to room temperature and precipitated three times with ethanol from toluene to remove excess OA. The size of the NPs is controlled by the Fe(CO)_5_:OA ratio. The average size of the monodisperse SPIONs was determined to be 9.2 nm *via* transmission electron microscopy (TEM) (Supplementary Figure S1) using the Pebbles software (Mondini et al., 2012). The inorganic fraction of the washed OA-coated SPIONs was determined to be 30.7% of the total weight of the sample (Supplementary Figure S2) by thermogravimetric analysis (TGA).

### Synthesis of the Bi-Functional Polymer

**FIGURE 2:**
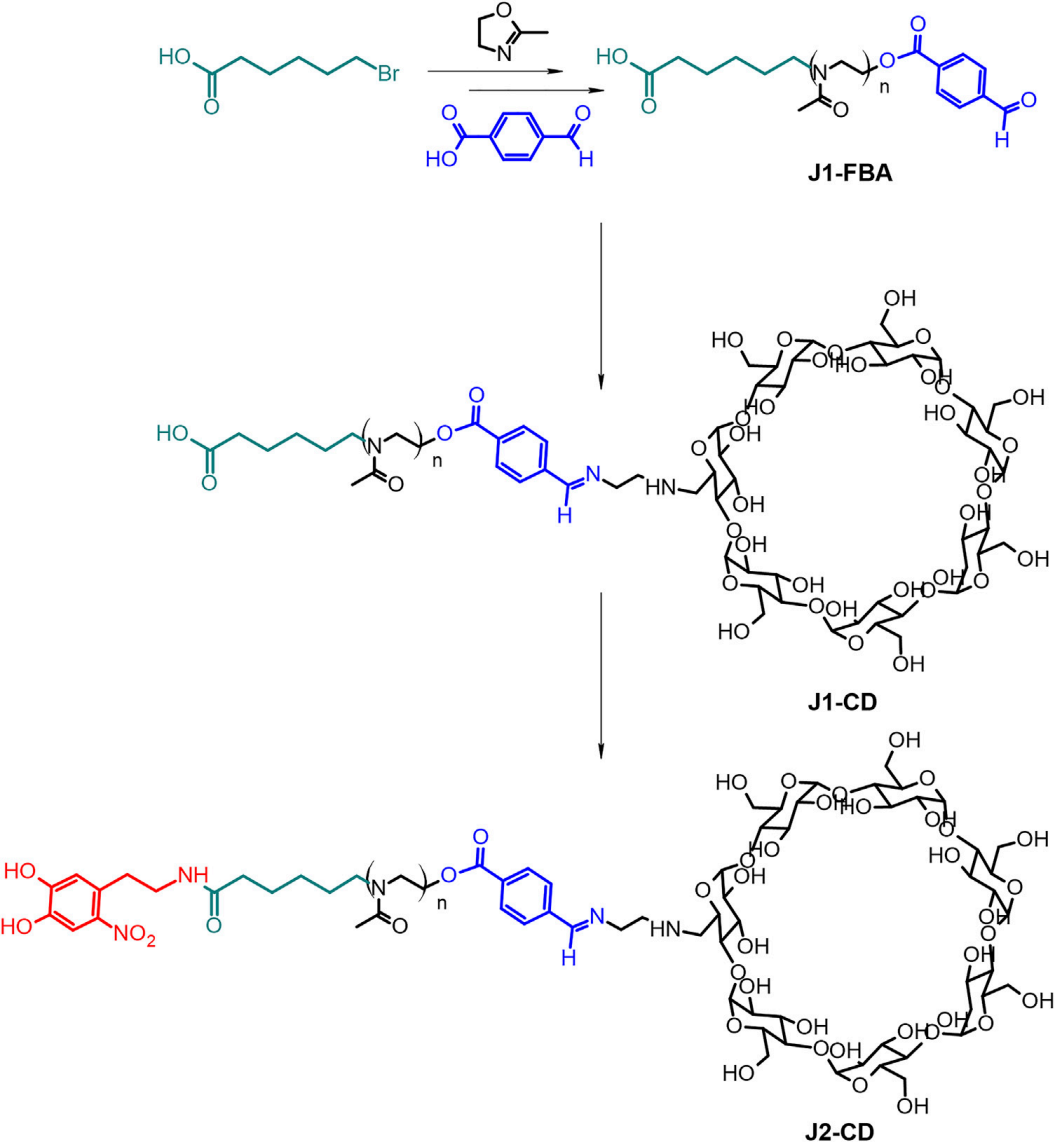
General scheme of synthesis of the bi-functional polymer.

The bi-functional polymer was prepared through a multi-step, modular synthesis described in the general synthetic scheme (Figure 2).

#### Synthesis of J1-FBA.

A linear poly (2-methyl-2-oxazoline) (PMOXA) bearing carboxylic and formylbenzil terminations at its two ends, respectively, (J1-FBA) was synthesized via CROP polymerization (Fijten et al., 2008) under a dry nitrogen atmosphere. 6-bromohexanoic acid (6BHA) 100 mg (0.513 mmol) was used as the initiator and reacted at 110°C with 8 ml of 2-methyl-oxazoline (94.5 mmol; x184) in 15 ml of anhydrous dimethylacetamide (DMA) for 22 h. The reaction mixture was then brought to 50°C and reacted with a 770 mg of 4-formylbenzoic acid (FBA, x10 excess) dissolved in 3 ml of anhydrous DMA in the presence of 2,6-lutidine (612 µL) for 22 h to terminate the reaction (Miyamoto et al., 1989). After this time, the solution was cooled to room temperature, and the polymer precipitated twice in diethyl ether (200 ml). Finally, the polymer was dialysed (cut off: 3.5 kDa) for 5 h and lyophilised to yield ∼4.5 g of J1-FBA.

^1^H NMR (DMSO): δ = 2.10 (247H, -C*H*_3_CO-), 3.45 (389H, -N-C*H*_2_-C*H*_2_-N-), 7.94 and 8.19 (4H, aromatics of the formylbenzoic group), 10.09 (1H, aldehyde).

NMR integration (Supplementary Figure S5) yielded a calculation of ∼85 repeating units for the polymer, which is ∼7,000 Da in molecular weight.

GPC: M_n_ 12,580, M*w* 23,300, M_w_/M_n_ 1.8 (Supplementary Figure S6).

#### Synthesis of J1-CD

A functional ethyl diamino-β-cyclodextrin (enCD) (see Supplementary Figures S3, S4 for its synthesis and characterization) was introduced to the polymer backbone by forming a benzoic imine. Such bonds are known to be stable at a neutral pH value (ca. 7.4) but start to hydrolyze at slightly acidic pHs.

1 g of J1-FBA (0.143 mmol) was reacted with 250 mg of enCD **(**see Supplementary Material **)** (0.213 mmol, 1:1.5 eq) in the presence of 50 µL of TEA in 10 ml of DMSO 50°C for 24 h. The polymer was then dialysed (cut off: 3.5 kDa) to remove DMSO and the excess of enCD to obtain ∼1 g of J1-CD.

^1^H NMR (DMSO): δ = 2.00 (194H, -C*H*_3_CO-), 3.75–3.00 (298H, -N-C*H*_2_-C*H*_2_-N-, 42H of βCD), 4.44 (6H, OH-6 of βCD), 4.84 (7H, anomeric-H of βCD), 6.20–5.60 (14H, OH-2 and OH-3 of βCD), 7.75–8.25 (4H, aromatics of the benzoic-imine group). (Supplementary Figure S7).

GPC: Mn 10,600, Mw 23,900, Mw/Mn 2.2 (Supplementary Figure S8).

FT-IR spectra of CD-terminated and aldehyde-terminated polymer are compared to support the chemical functionalization and formation of aryl-imine (Supplementary Figure S10). Changes in the IR absorption bands are observed in J1-CD due to the introduction of the CD’s hexoses; these are observed as significant increases in the OH and CH stretches (3,600–2,700 cm^−1^), the C-C/C-O stretching (1,130–900 cm^−1^), and the OH-torsions (broad background >800 cm^−1^) after CD coupling.

#### Synthesis of J2-CD

6-Nitrodopamine (NDA) was synthesized from 6-aminodopamine according to the literature (Supplementary Figure S11) (Napolitano et al., 1992). The nitrocatechol-terminated polymer was synthesised from 790 mg of the carboxy-terminated J1-CD (0.113 mmol) by dissolving in 10 ml anhydrous DMF in an inert atmosphere. Thereafter, 45 mg (0.14 mmol) TBTU and 24 μL DIPEA (0.12 mmol) were added and stirred for 15 min. NDA (33 mg, 0.14 mmol) was added as a solution in anhydrous DMF. The reaction solution was stirred in the dark for 24 h. The polymer was dialysed (cut off: 3.5 kDa) against water to remove DMF, the excess of NDA, and the coupling agents to yield ∼750 mg of J2-CD.

^1^H NMR (DMSO): δ = 2.00 (193H, -C*H*_3_CO-), 3.75–3.00 (290H, -N-C*H*_2_-C*H*_2_-N-, 42H of βCD), 4.44 (6H, OH-6 of βCD), 4.84 (7H, anomeric-H of βCD), 6.20–5.60 (14H, OH-2 and OH-3 of βCD), 7.30–7.50 (2H, aromatics of the NDA group), 7.75–8.25 (4H, aromatics of the benzoic-imine group). (Supplementary Figure S12).

### Ligand Replacement and Purification of Polymer-Coated Iron Oxide NPs

Core-shell SPIONs were obtained via ligand exchange, yielding monodisperse, colloidally stable, and biocompatible nanoparticles.

100 mg of wet NPs (from EtOH washing) (Supplementary Figures S1, S2) were dispersed in 10 ml of DMF together with 600 mg of J2-CD, representing a ∼20-fold excess with respect to the grafting density of 1 NDA-terminated polymer/nm (Martín et al., 2014). The dispersion was sonicated for 5 min at room temperature, and the mixture was shaken at 4°C for 6 days. After this time, the DMF suspension was precipitated with 25 ml of Et_2_O and centrifuged at 4000 RPM for 5 min. The supernatant was discarded, and the residue was washed twice with Et_2_O to remove residual DMF and free oleic acid, leaving a sticky precipitate containing the functionalized nanoparticles and the excess of free polymer.

The core-shell nanoparticles were then purified by dispersing the obtained solid residue in DI water and removing the excess of free polymer using centrifugal filters (Amicon^®^ Ultra-15 Centrifugal Filters, RC 30 kDa MWCO) at 3000 RPM for 15 min. The operation was repeated several times until the separated solution appeared clear of polymer.

## Results and Discussion

### pH-Responsiveness of the Polymer

**FIGURE 3:**
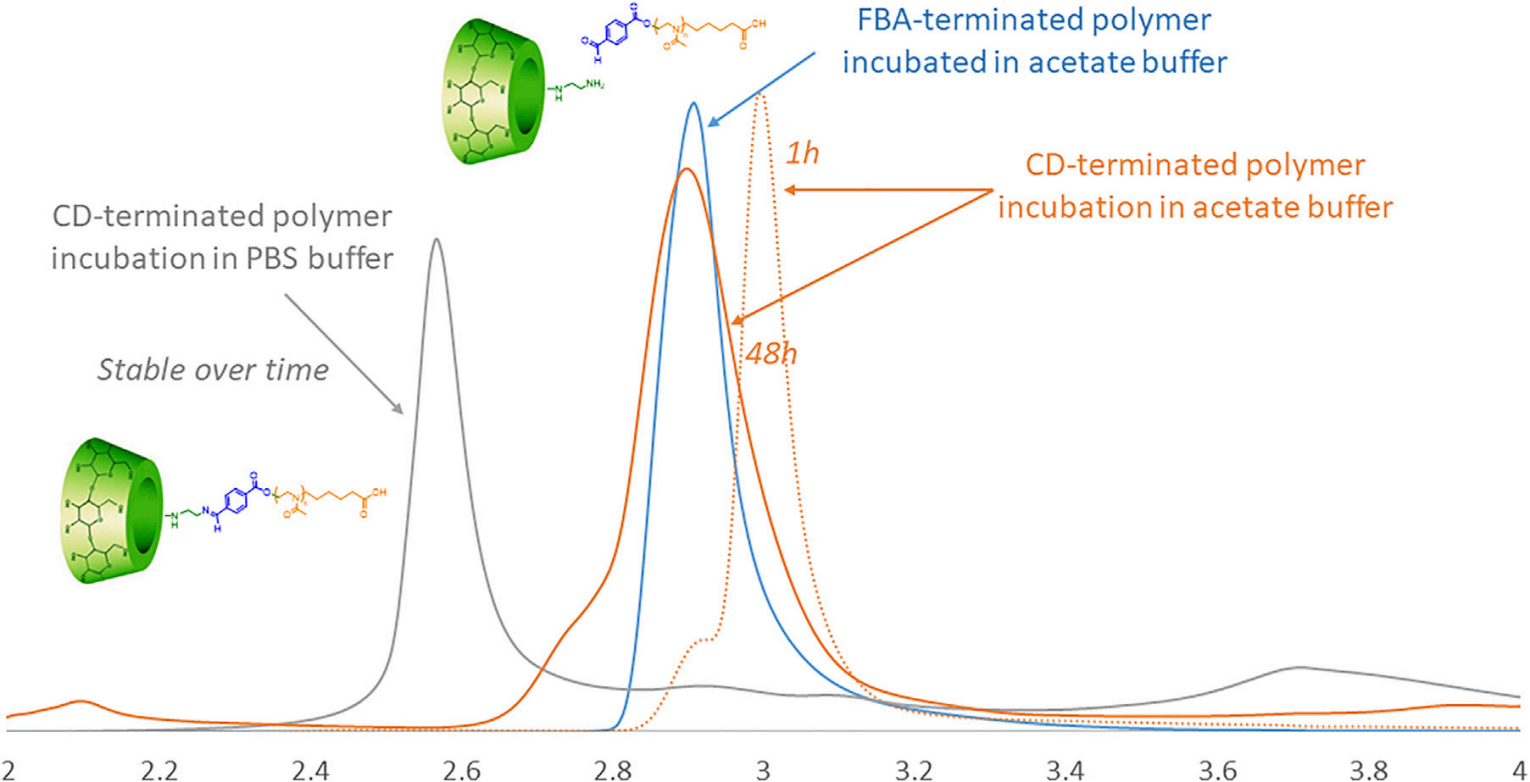
HPLC traces of CD-terminated polymer incubated in acetate over time (orange) in comparison to the same polymer in PBS (grey) and the reference FBA-terminated polymer incubated in acetate buffer (blue).

To assess the pH-triggered hydrolysis of the CD in the polymer, we studied the chromatographic profiles via HPLC of J1-CD in acetate buffer (pH 5.5) and in PBS (pH 7.4) over time (Figure 3). The HPLC ramp was 15–25% (acetonitrile/water) in 30 min with a flow rate of 0.6 ml/min on a HyperClone 250 × 4.60 mm, 5 micron column. The detection of the fractions was performed by UV at 256 nm. Figure 3 shows that the CD-terminated polymer incubated in PBS (pH 7.4) elutes as a single peak (grey curve), demonstrating that the polymer is stable at biological pH. The elution peak does not change with incubation time.

Incubation in acetate buffer should, over time, lead to the release of CD linked to the PMOXA via the pH-sensitive FBA linker, thereby changing the elution time. The light yellow elution profile shows the J1-CD in acetate as prepared. After 48 h incubation in PBS buffer (pH 7.4) there was no change is the retention time for J1-CD, whilst after the same time in acetate buffer at pH 5.5, the elution peak has changed to the same position as the J1-FBA polymer in acetate buffer, thereby demonstrating that the CD macrocycle has been cleaved off.

### CySPION Characterization

Through thermogravimetric analysis (TGA), the inorganic fraction for purified CySPIONs was determined to be 12.2% of the total weight of the sample (Supplementary Figure S13). The grafting density was calculated to be ∼2.5 NDA-PMOXA/nm (Martín et al., 2014), which is a very high grafting density compatible with an expectation of excellent colloidal stability in biofluids (Zirbs et al., 2015). Based on the number of CD-bearing polymer chains per squared nm and knowing the surface of the magnetite nanoparticle, we can estimate the total number of CDs attached on CySPION surface to be around 700.

The CySPION hydrodynamic size was determined using dynamic light scattering (DLS) in PBS buffer at 1.0 mg/ ml, revealing an average hydrodynamic diameter of 77 nm by number- and volume-weighted distributions alike (PDI 0.217) (Supplementary Figure S14). The obtained hydrodynamic diameter agrees with the expectations for SPION grafted with M_w_ 23 kDa of PMOXA (by GPC) and functionalised with CD (Kurzhals et al., 2017). Obtaining the same size for both distributions demonstrate a highly monodisperse colloidal sample. The dispersions of CySPIONs in DI water and PBS buffer were stable upon storage at room temperature for more than 10 days, the length of time we investigated it, indicating chemical stability of the nanoparticles under physiological ionic strength.

### Cholesterol-Mopping Activity Study of the CySPION

We assessed the CySPION’s capacity to sequester cholesterol via a Cholesterol Assay Kit (Sigma-Aldrich MAK043) (dos Santos et al., 2011) in acetate buffer. The assay quantifies the cholesterol concentration through a coupled enzyme assay, resulting in a fluorometric measurement (λ_ex_ = 535 nm, λ_em_ = 595 nm) proportional to the cholesterol present. Briefly, a cholesterol stock solution was prepared by dissolving 10 mg of cholesterol powder in ethanol (1 ml). 15 µL of the stock (representing an excess amount, that is, 1.6x, 3.2x, 6.4x, 31x excess in mols, respectively, compared to the estimated concentration of CDs in the CySPION samples) was then added to a 1 ml solution in acetate buffer containing increasing concentrations (0–2 mg/ ml) of CySPION and stirred for 72 h at 37°C. The samples were then centrifuged (5000 RPM, 4500 RCF, for 2 h at 4°C) and subsequently filtered through a 0.45-μm regenerated cellulose syringe filter to remove the excess of insolubilized cholesterol and the nanoparticles.

In therapy, the therapeutic effect would be produced by CySPION taken up by cells, entering the endosome, and releasing CD cleaved off at the successively lower pH as the endosome progresses to the lysosome. Our experiment tests the desired functionality by analyzing the efficiency by which released CD solubilizes cholesterol by forming inclusion complexes. After centrifugation and filtration, only cholesterol in inclusion complexes with the CD is present in the supernatant, i.e., the active part of our therapeutic nanoparticle platform.

**FIGURE 4:**
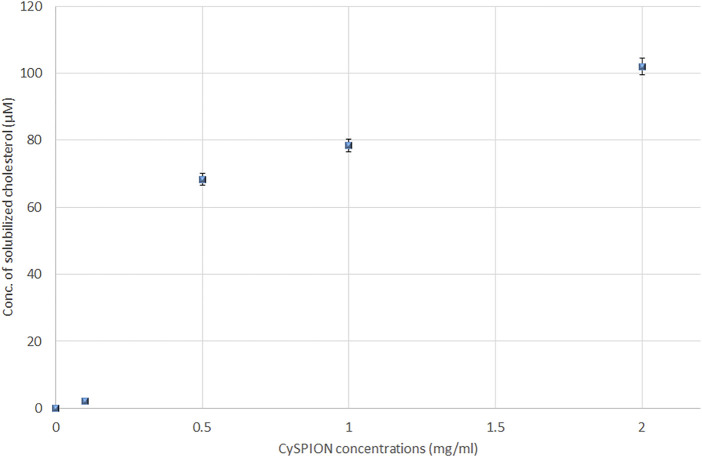
Solubilized cholesterol (in µM) due to the formation of inclusion complex with β-CD from hydrolysis of CySPION in Acetate buffer.

The concentration of solubilised cholesterol was measured for each sample at pH 5.5 using acetate buffer following the Assay Kit’s instructions. Figure 4 shows an increase in the amount of solubilised cholesterol with the CySPION concentration. As expected, the detected solubilised cholesterol increases with the CySPION concentration. The calculated ratio between the solubilised cholesterol and the estimated CD concentration on the CySPION is close to 1:2 for the 2.0 and 1.0 mg/ ml samples and close to 1:1 in the 0.5 mg/ ml, which is in line with the modelled cholesterol-CD inclusion complex stochiometries (López et al., 2011). Based on the pH-responsiveness test, we expect all the CDs to have been released from the CySPION after the incubation in acetate buffer and be active in sequestring cholesterol from solution. No significant amount of cholesterol was solubilised with the incubation at 0.1 mg/ ml and no cholesterol was detectable in the control (that is, without CySPION) in acetate buffer. At physiological pH using PBS buffer no cholesterol was detected following the same protocol as the CySPION is stable at this pH, showing that no CDs capturing cholesterol were released into the supernatant, as expected.

## Conclusion

We demonstrated the synthesis and characterization of a novel, CD-decorated, and colloidally stable nanoparticle system (CySPION) based on a SPION core and a functional PMOXA shell. Its nano-architecture was engineered so that its CD load could be released at slightly acidic pHs, suitable for lysosomal delivery and therapy. Our CySPION convincingly showed cholesterol-mopping activity through an enzymatic assay at lysosomal pH. The packaging of CDs into a nano-architecture such as CySPION and its smart pH-triggered delivery offer a significant advantage over the current administration of CDs in its monomeric form, which suffers from several drawbacks regarding reaching the therapeutic target and avoiding side effects. Additionally, the CySPIONs could improve BBB crossing and bioavailability. Therefore they represent a promising therapeutic tool for cholesterol-associated diseases such as neurodegenerative diseases and some rare genetic disorders, which we will investigate in future work.

## Data Availability

The original contributions presented in the study are included in the article/Supplementary Material, further inquiries can be directed to the corresponding author.
