# Supplementary material for: Cyclodextrin-Appended Superparamagnetic Iron Oxide Nanoparticles as Cholesterol-Mopping Agents
Source: Front Chem. 2021 Nov 18;9:795598. doi: 10.3389/fchem.2021.795598 (PMC8636776; doi:10.3389/fchem.2021.795598)
Supplement: Supplementary file 1 [file DataSheet1.docx]

Cyclodextrin-appended superparamagnetic iron oxide nanoparticles as cholesterol-mopping agents

Antonino Puglisi^1*^, Simone Bassini^1,2^, Erik Reimhult^1^

^1^Institute of Biologically Inspired Materials, Department of Nanobiotechnology, University of Natural Resources and Life Sciences (BOKU), Vienna, Austria

^2^ Life Sciences Department, University of Modena and Reggio Emilia, Italy

*** Correspondence:**Antonino Puglisi
[antonino.puglisi@boku.ac.at](mailto:antonino.puglisi@boku.ac.at)


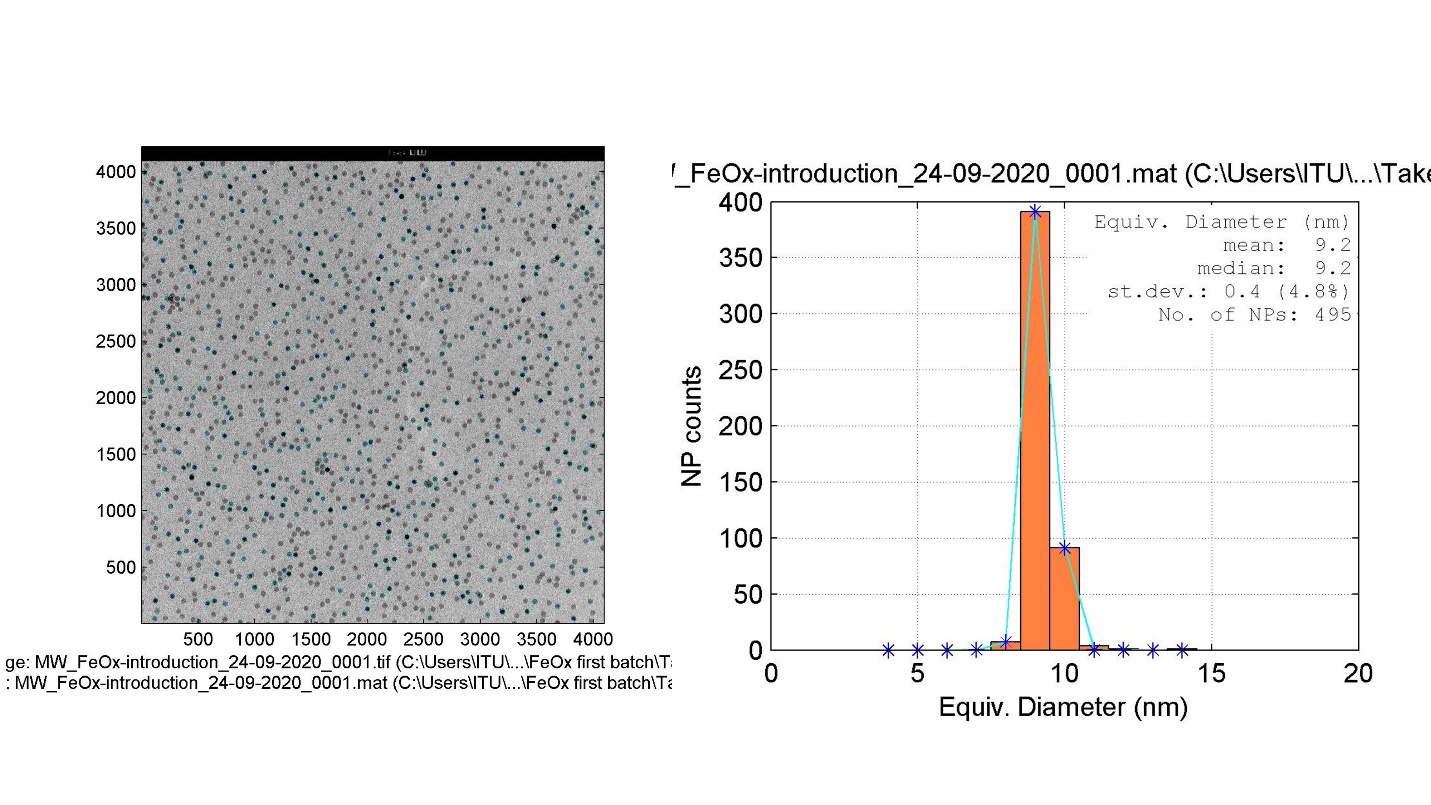
Figure S1 - TEM image and size distribution calculation for OA-coated nanoparticles


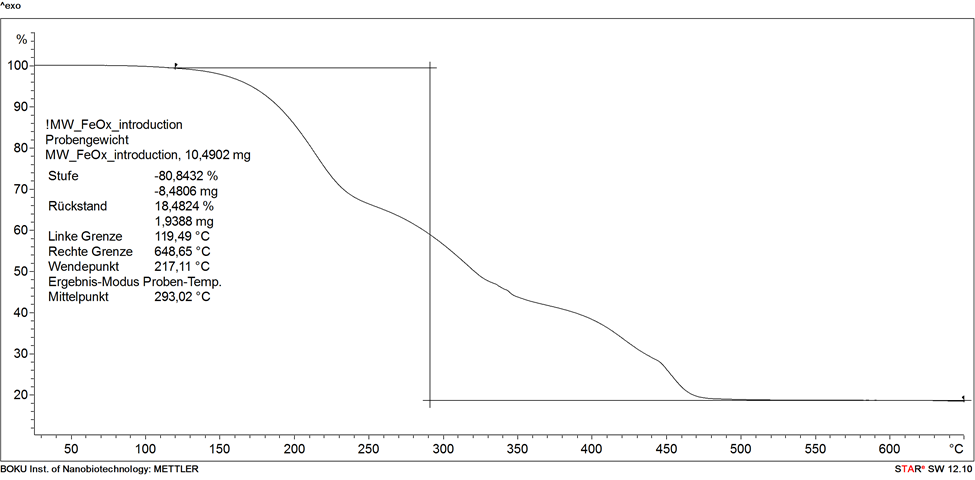


Figure S2 - TGA of OA-coated nanoparticles

*Synthesis of ethyl diamino-β-cyclodextrin (enCD).* Functional ethyl diamino-β-cyclodextrin (enCD) bearing a primary amino group for further polymer modification was synthesized from mono-6-(p-tosyl-sulfonyl)-β-cyclodextrin (CD-Tos) (Sigma-Aldrich) and ethylenediamine (EDA) as described previously^[[1]](#footnote-1)^. CDTos (1.0 g; 0.776 mmol) was added into 15 mL of EDA and stirred at 80°C overnight. After removing the unreacted EDA by a rotary evaporator, the residues were dissolved in water and purified by repeated precipitation into acetone. The final product enCD was dried in a vacuum oven overnight at room temperature obtaining 890 mg (97%). ESI-MS: m/z 1178 (S3).


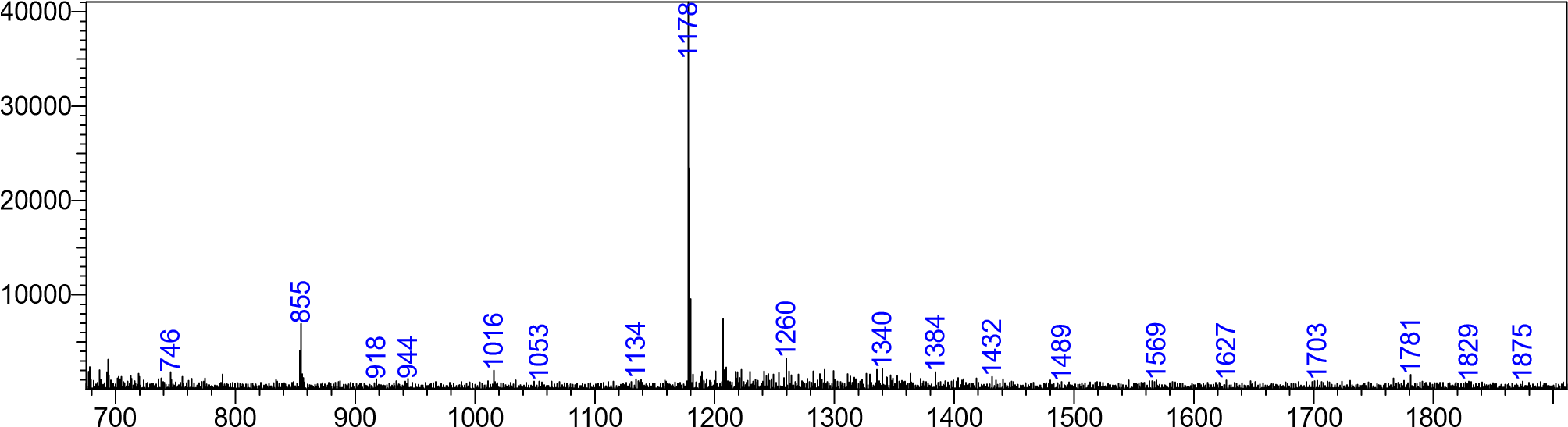


Figure S3 - ESI-MS of enCD

**
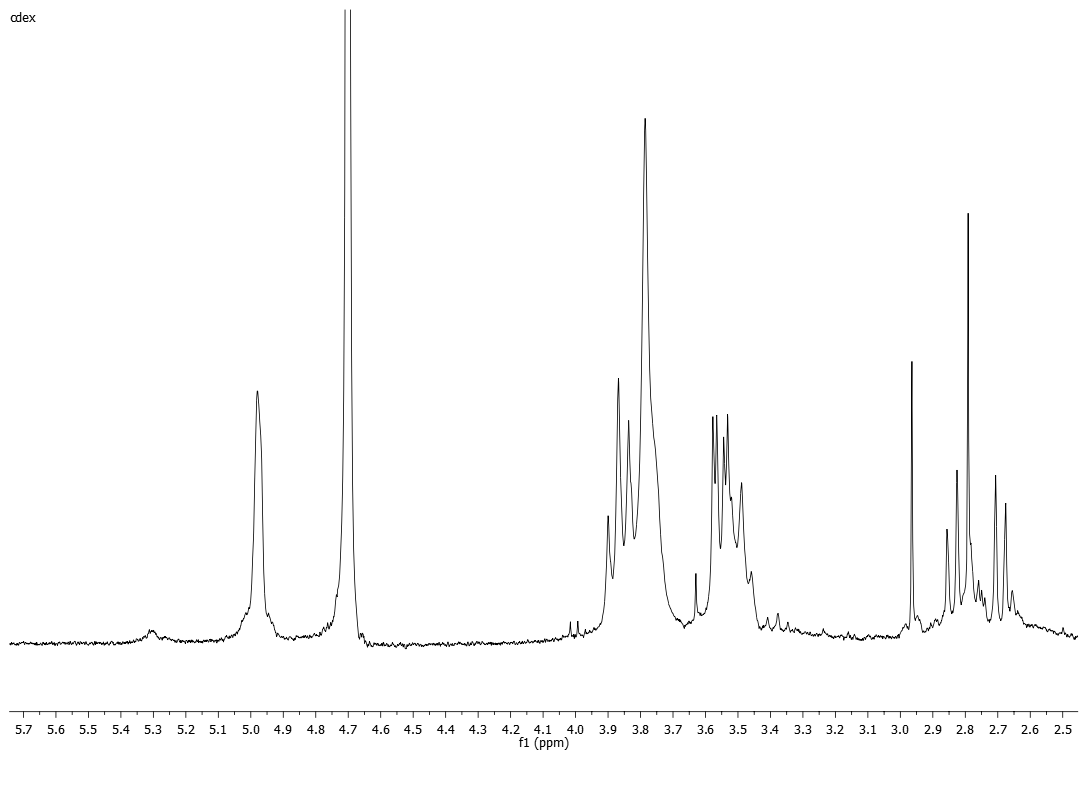
**

Figure S4 - ^1^H-NMR spectrum (300 MHz, D_2_O, 25°C) of enCD

FBA-terminated poly(2-methyl-2-oxazoline) (J1-FBA)


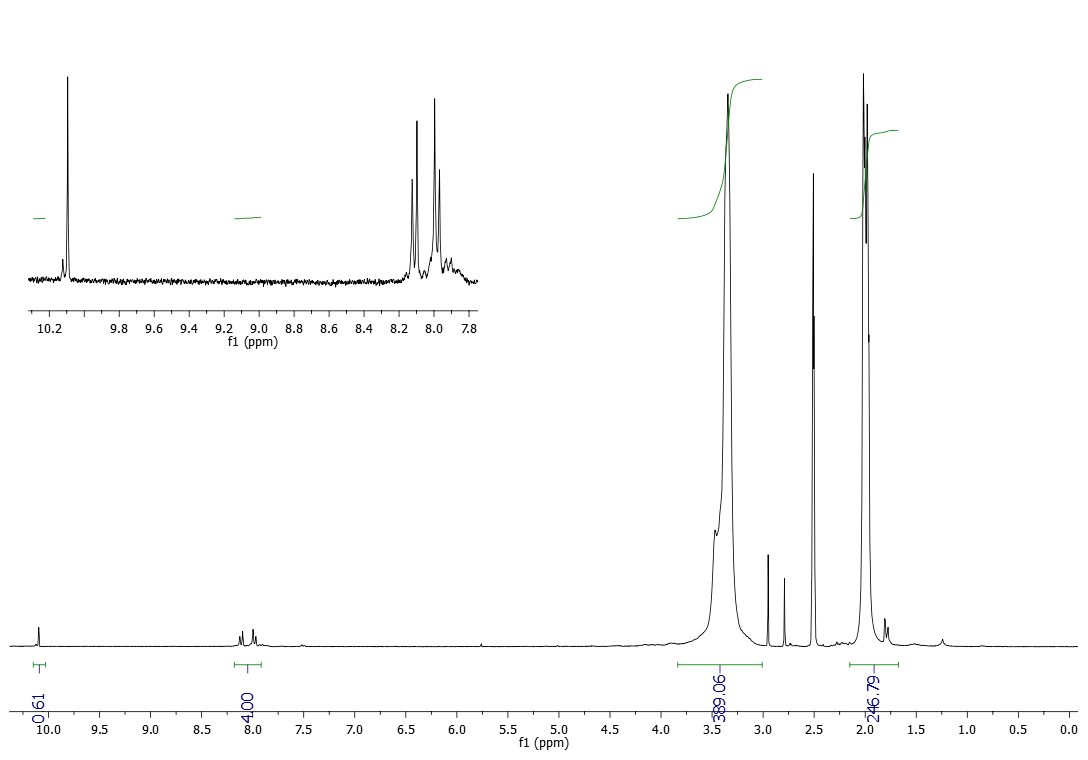


Figure S5 - 1H-NMR spectrum (300 MHz, DMSO, 25°C) of J1-FBA

Figure S6 - GPC of J1-FBA

M_n_ 12,580, M*w* 23,300, M_w_/M_n_ 1.8

CD-terminated poly(2-methyl-2-oxazoline) (J1-CD)


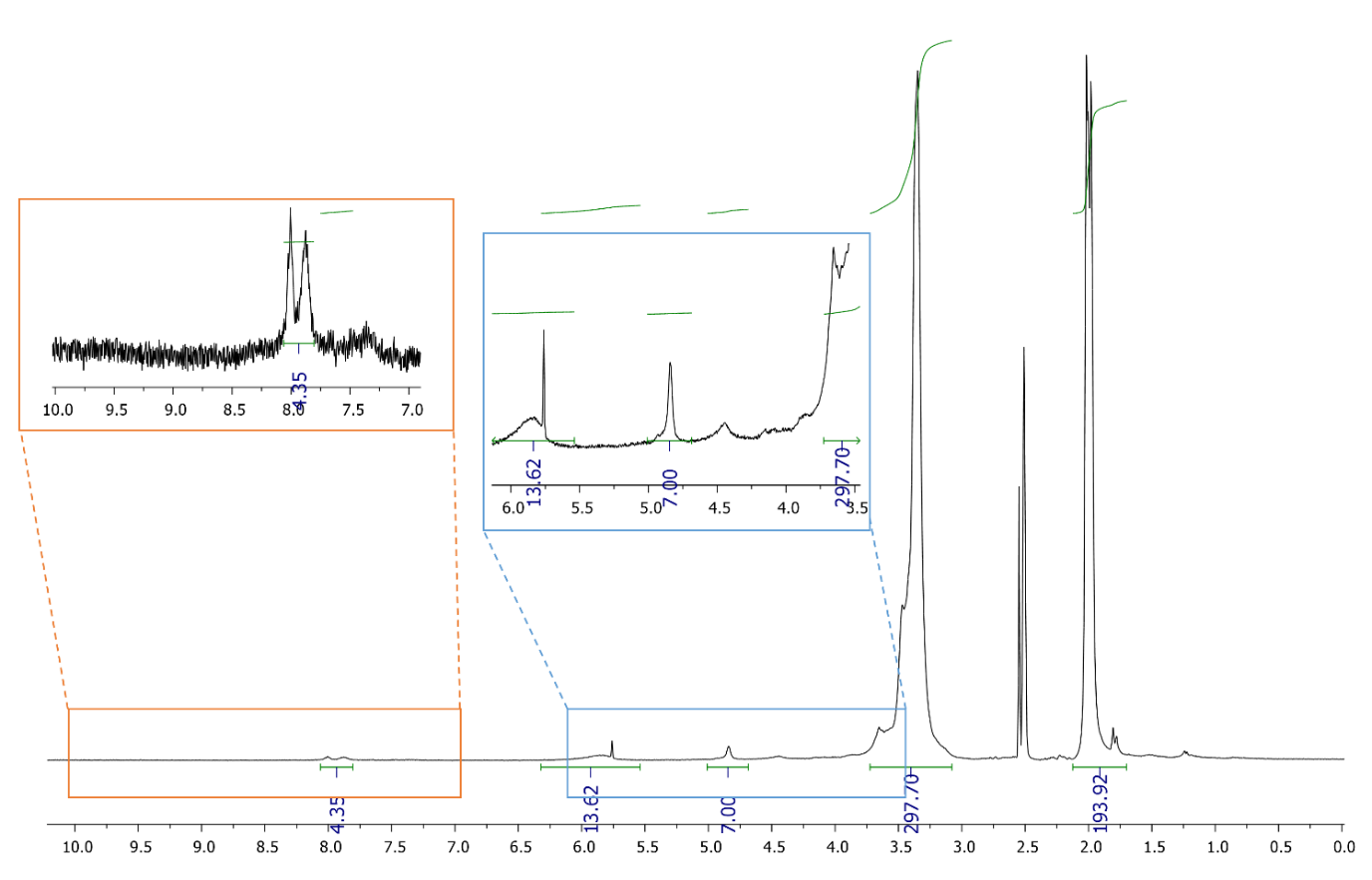


Figure S7 - ^1^H-NMR spectrum (300 MHz, DMSO, 25 °C) of J1-CD

Figure S8 - GPC of J1-CD

M_n_ 10,600, M*w* 23,900, M_w_/M_n_ 2.2


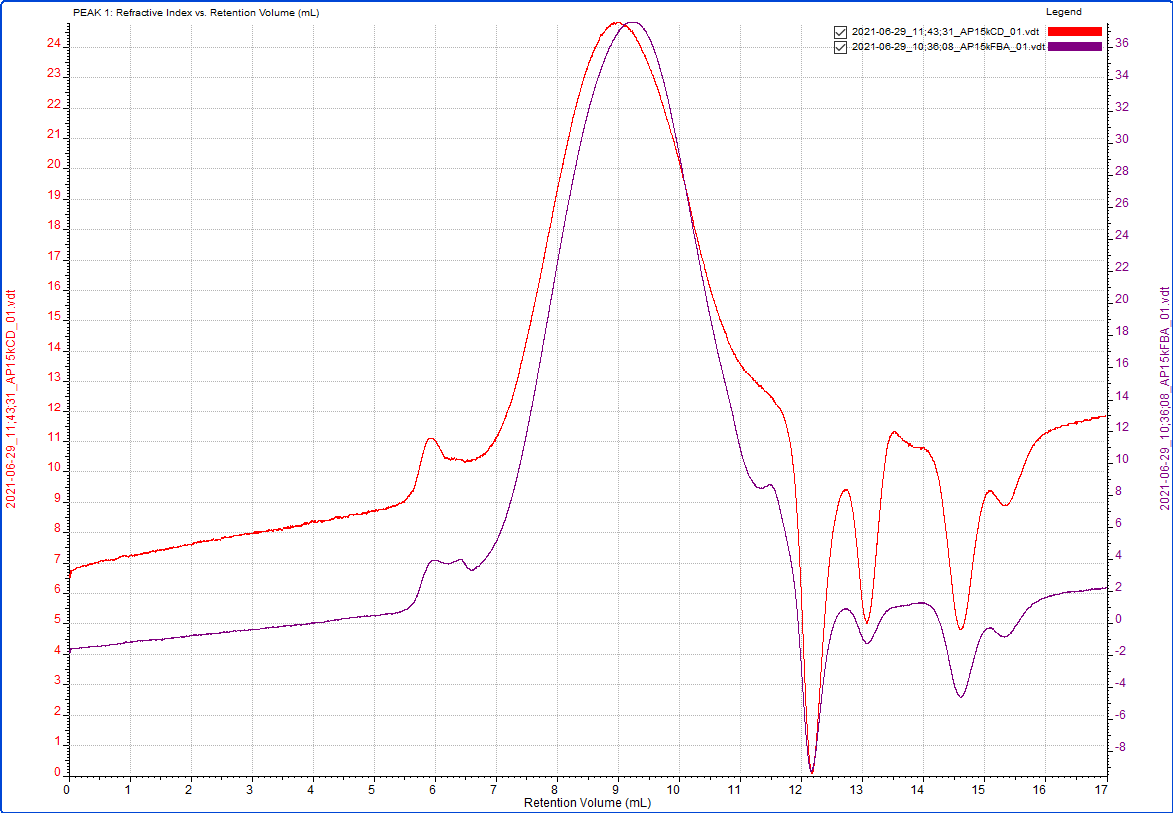


Figure S9 - GPC overlay of J1-FBA and J1-CD


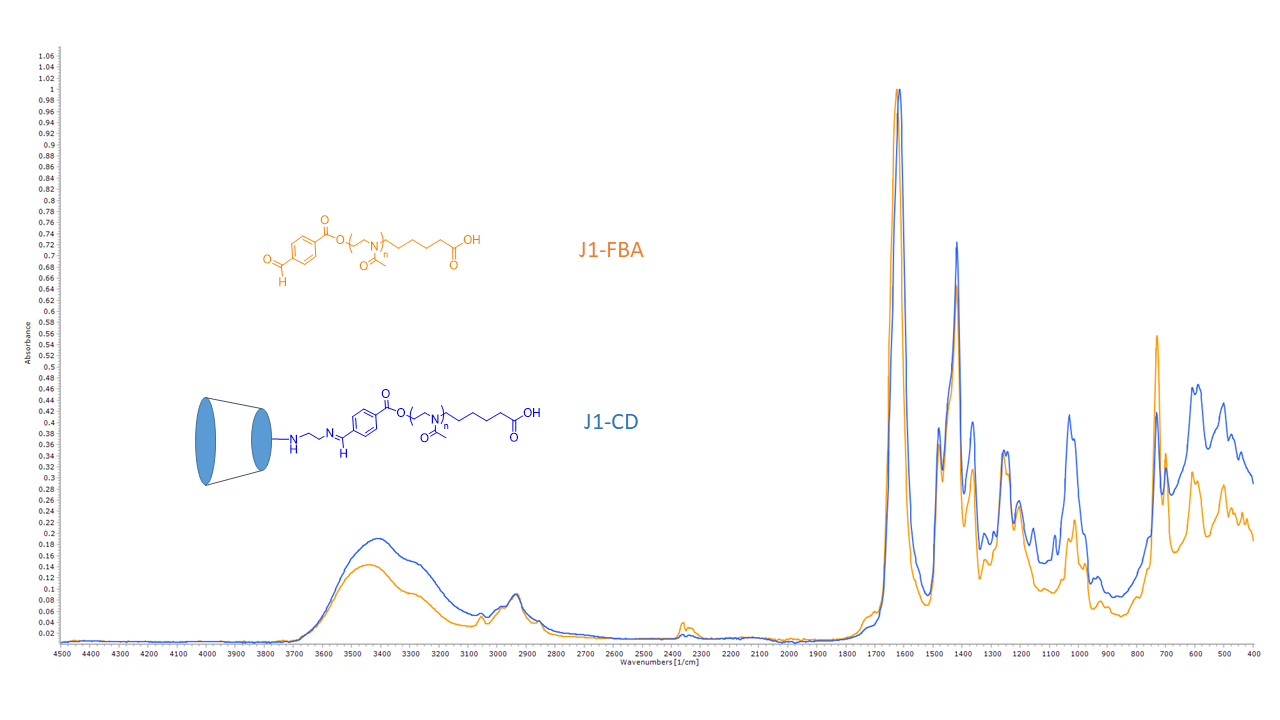


Figure S10 - IR-spectra for J1-FBA (orange) and J1-CD (in blue)


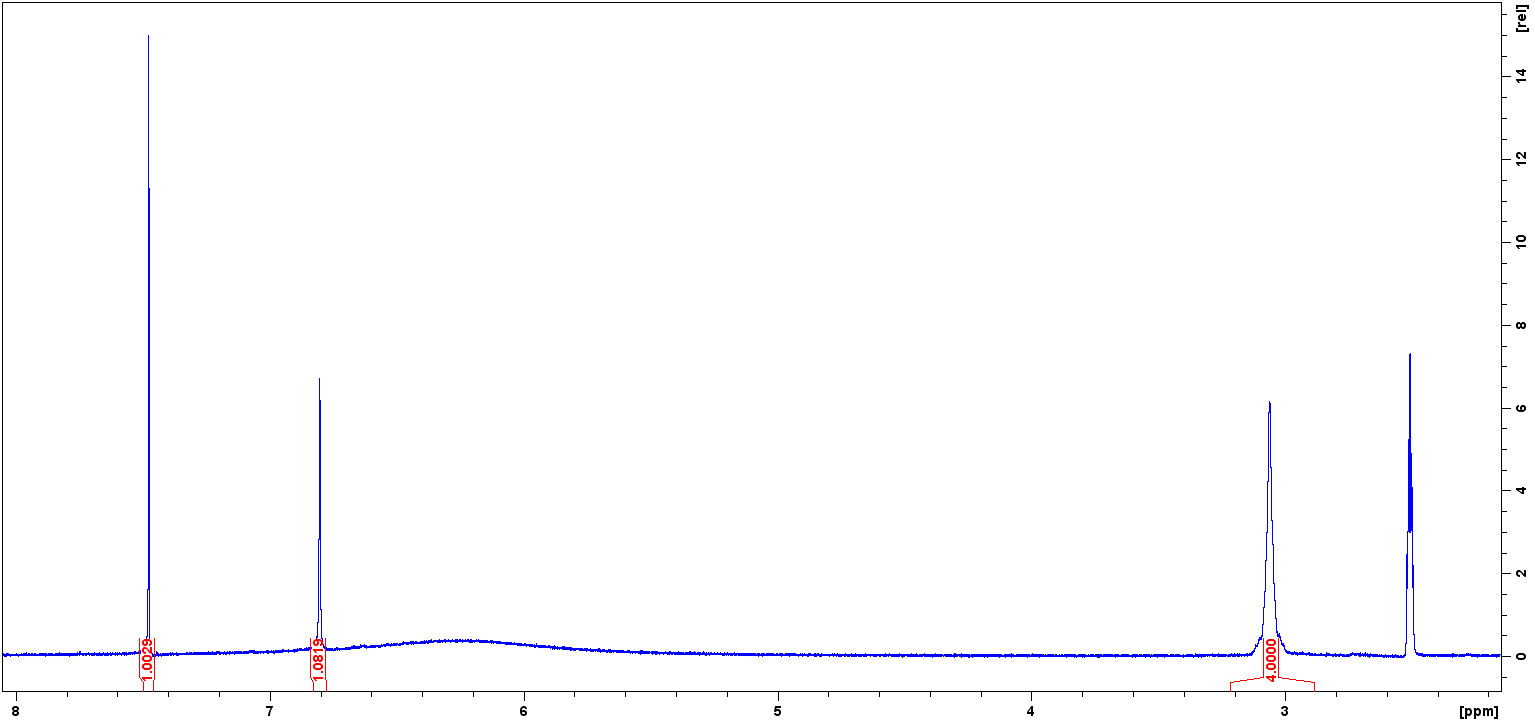


Figure S11 - ^1^H-NMR spectrum (300 MHz, DMSO, 25 °C) of NDA


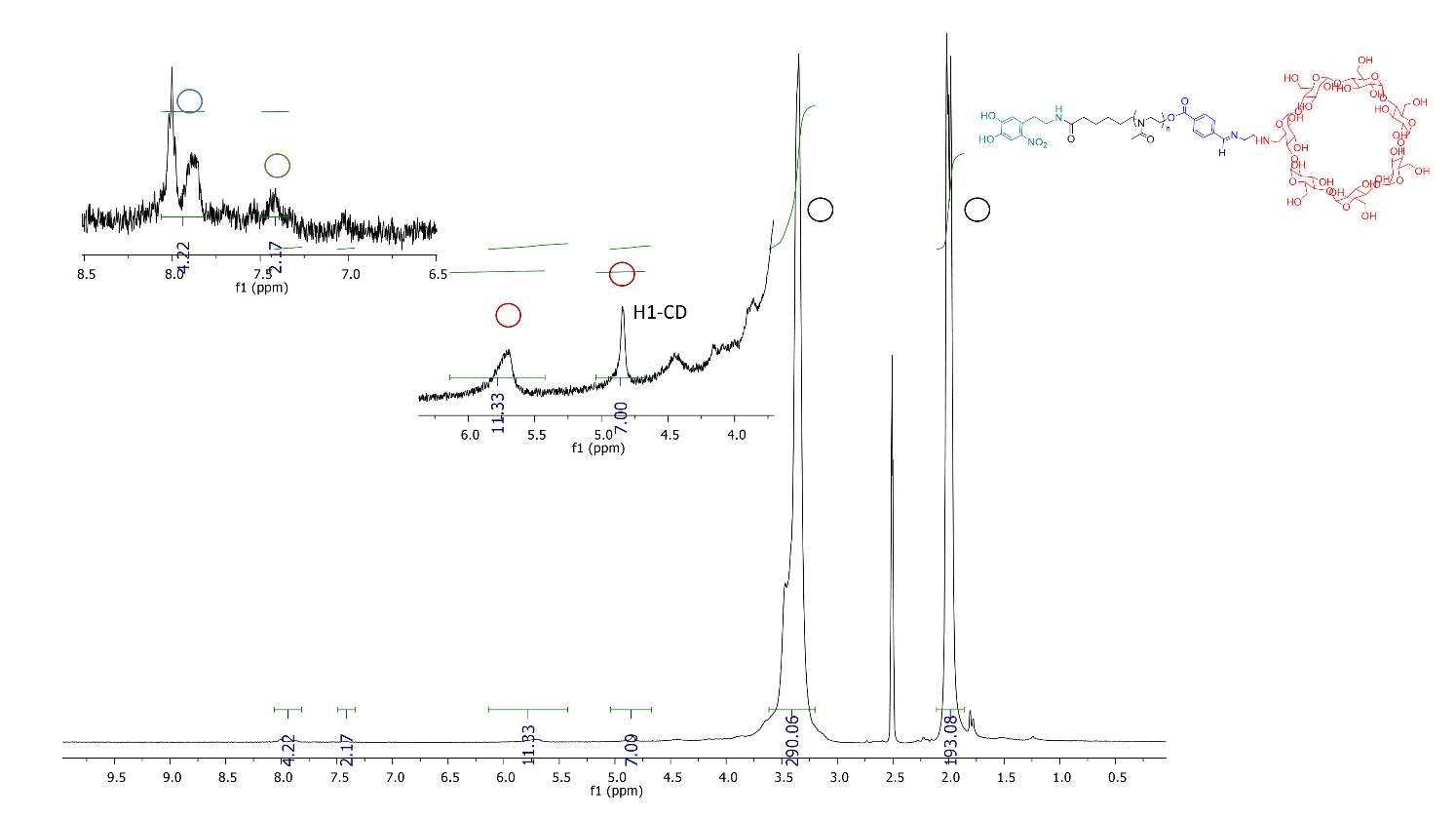


Figure S12 - ^1^H-NMR spectrum (300 MHz, DMSO, 25 °C) of J2-CD


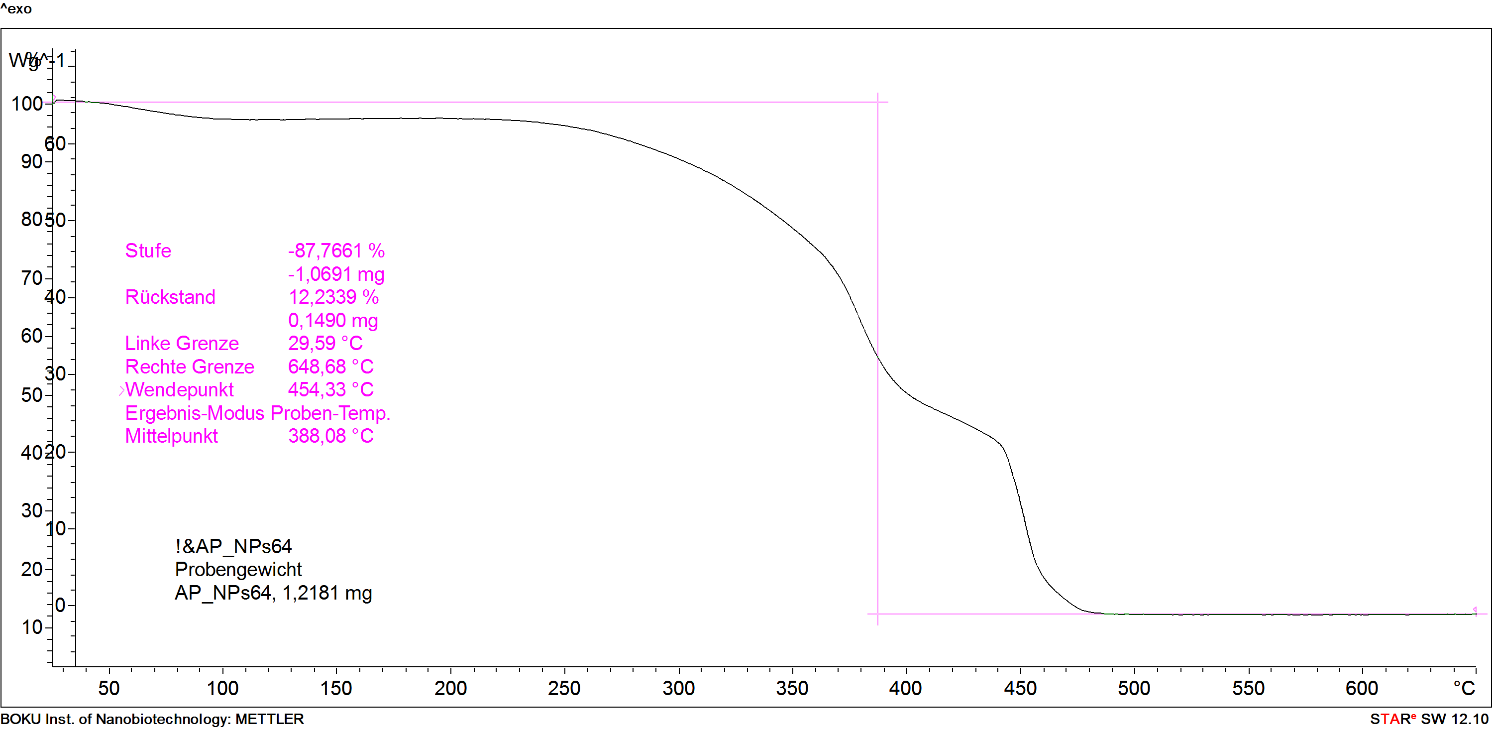


Figure S13 - TGA of polymer-coated nanoparticles


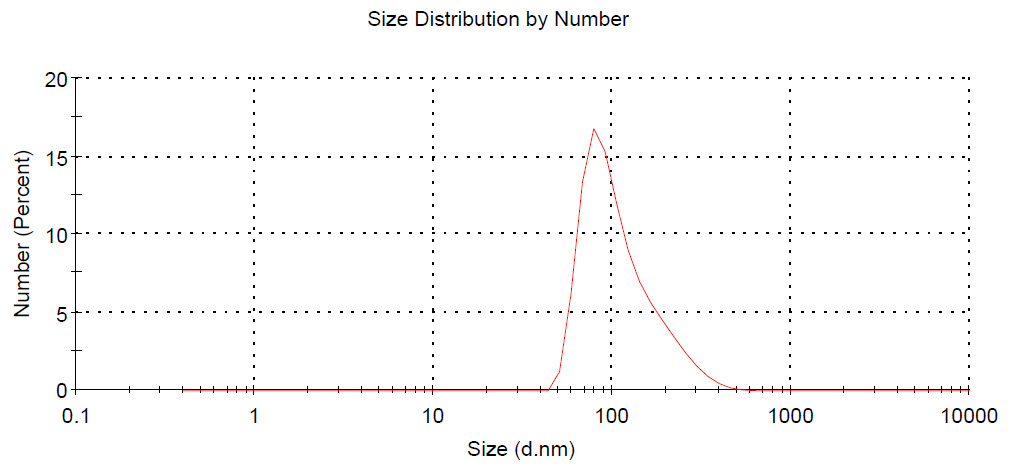


Figure S14 – Number-weighted DLS size distribution for CySPION at 1 mg/mL in PBS (77 nm)

1. Liu, Y. Y., Fan, X. D. & Gao, L. J. M. B. Synthesis and Characterization of β‐Cyclodextrin Based Functional Monomers and its Copolymers with N‐isopropylacrylamide. 3, 715-719 (2003) [↑](#footnote-ref-1)
